# Sensitivity and specificity evaluation of multiple neurodegenerative proteins for Creutzfeldt-Jakob disease diagnosis using a deep-learning approach

**DOI:** 10.1080/19336896.2019.1639482

**Published:** 2019-07-15

**Authors:** Sol Moe Lee, Jae Wook Hyeon, Soo-Jin Kim, Heebal Kim, Ran Noh, Seonghan Kim, Yeong Seon Lee, Su Yeon Kim

**Affiliations:** a Division of Bacterial Disease Research, Center for Infectious Diseases Research, Korea National Institute of Health, Centers for Disease Control and Prevention, Cheongju-si, Chungcheongbuk-do, South Korea; b Department of Agricultural Biotechnology and Research Institute of Agriculture and Life Sciences, Seoul National University, Seoul, South Korea

**Keywords:** Deep learning, Tau, α-synuclein, 14-3-3, β-amyloid, prion diseases, Creutzfeldt-Jakob disease

## Abstract

The diagnosis of sporadic Creutzfeldt-Jakob disease (sCJD) can only be confirmed by abnormal protease-resistant prion protein accumulation in post-mortem brain tissue. The relationships between sCJD and cerebrospinal fluid (CSF) proteins such as 14–3-3, tau, and α-synuclein (a-syn) have been investigated for their potential value in pre-mortem diagnosis. Recently, deep-learning (DL) methods have attracted attention in neurodegenerative disease research. We established DL-aided pre-mortem diagnostic methods for CJD using multiple CSF biomarkers to improve their discriminatory sensitivity and specificity. Enzyme-linked immunosorbent assays were performed on phospho-tau (p-tau), total-tau (t-tau), a-syn, and β-amyloid (1–42), and western blot analysis was performed for 14–3-3 protein from CSF samples of 49 sCJD and 256 non-CJD Korean patients, respectively. The deep neural network structure comprised one input, five hidden, and one output layers, with 20, 40, 30, 20 and 12 hidden unit numbers per hidden layer, respectively. The best performing DL model demonstrated 90.38% accuracy, 83.33% sensitivity, and 92.5% specificity for the three-protein combination of t-tau, p-tau, and a-syn, and all other patients in a separate CSF set (n = 15) with other neuronal diseases were correctly predicted to not have CJD. Thus, DL-aided pre-mortem diagnosis may provide a suitable tool for discriminating CJD patients from non-CJD patients.

## Introduction

Creutzfeldt-Jakob disease (CJD) is the most common human prion disease, which is characterized by the abnormal accumulation of misfolded prion protein (PrP^Sc^), thus affecting the central nervous system [1,2]. The global incidence of CJD diagnosis is approximately 1 per million individuals per year. The most common form is sporadic CJD (sCJD), which accounts for about 85–95% of all known cases.

A definitive diagnosis of sCJD requires histological analysis of brain tissues obtained during autopsy [3], but it cannot always clinically distinguish CJD from other rapidly progressive neurodegenerative disorders in living patients, since these diseases share several clinical characteristics [4–6]. Therefore, there has been extensive research effort devoted to identifying cerebrospinal fluid (CSF) protein biomarkers for the pre-mortem diagnosis of sCJD [7–10]. Indeed, diagnosis using CSF biomarkers has shown several benefits of reducing the diagnostic turn-around time with reproducible data without any risk of damage to the patient’s brain. One of these candidate biomarkers for sCJD is the 14–3-3 protein, showing diagnostic sensitivities ranging from 61% to 96% and specificities ranging from 67% to 95% [11–16]. Moreover, quantification of the CSF protein α-synuclein (a-syn) using an in-house enhanced chemiluminesence-based enzyme-linked immunosorbent assay (ELISA) was recently reported to be an excellent approach for sCJD diagnosis [10,17]. The total tau (t-tau) and phospho-tau (p-tau) levels, along with the p/t-tau ratio have also been suggested as clinically useful diagnostic markers [18,19]; however, these CSF biomarkers have not shown significantly high sensitivity or specificity in our experience with Korean patients with CJD, which is a major limitation of these biomarkers for this population.

An alternative diagnosis method is a real-time quaking-induced conversion (RT-QuIC) assay, which is a prion protein conversion assay that allows for the detection of the abnormal amyloid form of PrP, and has been reported to exhibit high sensitivity and specificity for sCJD in the range of 76.5–97% and 99–100%, respectively [20–23].

Machine learning (ML) and deep learning (DL), which are subfields of AI, are powerful tools for extracting patterns and characteristics from big data using neural networks [24–26]. In contrast to traditional ML, DL uses multiple neural networks with many hidden layers. For the past decade, DL had remarkable successes in various fields such as computer vision [27,28], natural language processing [29,30], and audio signal processing [31]. Recently, it has gained attention for disease diagnosis owing to their good representational power [32], including research on CJD [33–35]. Specifically, Morabito et al. [35] reported that a DL-based analysis, which used electroencephalography signals, showed 89% accuracy in discriminating between CJD patients and patients with other forms of rapid progressive dementia, with 92% sensitivity and 89% specificity.

The aims of the present study were to establish a pre-mortem diagnostic method using a DL approach, which would (1) facilitate diagnostic decision making for CJD, (2) improve accuracy compared to conventional pre-mortem diagnostic methods using CSF protein markers, and (3) help to identify more CJD-related CSF biomarkers as landmarks. We then tested the ability of the established DL technique to discriminate CJD patients from non-CJD patients based on detection levels of the key CSF biomarkers 14–3-3, a-syn, amyloid beta (Aβ), t-tau, and p-tau.

## Materials and methods

### Subjects

We collected CSF samples from 49 patients with sCJD (5 definite and 44 probable) and 11 with possible sCJD, and 256 non-CJD patients (these cases were referred to as ‘‘suspected CJD” to the KNIH, but were not confirmed as CJD) ascertained through routine surveillance by the Korea National Institute of Health (KNIH) according to diagnostic criteria (Table 1). Sequencing of all patient samples revealed a methionine residue at codon 129 of the prion protein-coding gene PRNP (129M/M homozygotes). These samples, except for those of 11 patients with possible sCJD, were used as the training, validation, and test sets (test set_A) for ML and DL analysis. In total, 15 CSF samples from patients who were diagnosed with other types of neuronal diseases were also collected and used as a separate test set (test set_B).

**Table 1.** Patients’ classification and clinical features of the definite, probable, possible CJD cases and other neuronal diseases patients used in this study.

Groups	No. of patients	Diagnostic criteria/Symptoms
Definite CJD	5	Diagnosed by neuropathological techniques, immunohistochemcal detection of PrP^Sc^ of autopsied or biopsied brain tissues from patients with probable or possible sCJD
Probable CJD	44	Rapid progressive dementia of less than 2 years’ duration and at least two of the following features such as myoclonus, visual or cerebellar disturbance, pyramidal, extrapyramidal dysfunction, akinetic mutism, with a EEG data, and/or a positive result of 14–3-3 assay
Possible CJD	11	Progressive dementia and at least two of the following features such as myoclonus, visual or cerebellar disturbance, pyramidal, extrapyramidal dysfunction, akinetic mutism, without a EEG data, and/or a positive result of 14–3-3 assay
Non-CJD	256	Cases referred to as ‘‘suspected CJD” to the KNIH, but were not fulfilling the criteria of probable or possible CJD
Neuronal diseases (test set_B)	3	Hydrocephalus, unspecified
	4	Normal Pressure Hydrocephalus
	1	Multi-system degeneration
	1	Alzheimer’s Disease
	1	Mild cognitive disorder
	1	Dementia
	1	Spinal muscular atrophy, and related syndromes
	1	Cerebral palsy
	2	Other disorders of brain

This study was approved by the Institutional Review Board (IRB) of the Korea Centers for Disease Control and Prevention (IRB No. 2017–03-09-C-A), and all the experiments were performed in accordance with relevant guidelines of the IRB. Written informed consent was obtained from the patients or their legal guardians.

### Biochemical analysis

The 14–3-3 protein levels in the CSF samples from the patients were determined by western blot analysis as previously described [8]. Quantitative determination of CSF p-tau, t-tau, Aβ, and a-syn proteins was conducted with the INNOTEST® hTAU Ag (Fujirebio, Gent, Belgium), INNOTEST® PHOSPHO-TAU(181P) (Fujirebio, Gent, Belgium), INNOTEST® β-AMYLOID(1–42) (Fujire-bio, Gent, Belgium), and SensoLyte Anti-α-Synuclein (Human, Mouse, Rat) ELISA Kit (AnaSpec, Inc., Fremont, CA), respectively, according to the manufacturers’ instructions. The p-tau to t-tau ratio (p/t-tau ratio) was also calculated based on these results.

### Data handling and scoring

To standardize the quantitative data of protein levels for the different candidate biomarkers, a positive band of 14–3-3 protein in the western blot was given a score of 2, whereas a negative or weakly positive finding was given a score of 1. When the a-syn or Aβ concentration was below the detection limit of the ELISA kits (7.813 ng/μl or 63 ng/μl, respectively), the case was given a score of 1.

To resolve the detrimental effect of imbalanced data on the performance of DL algorithms, we adopted an ‘oversampling’ approach [36–38]. Oversampling was performed after splitting the original dataset into the ‘training with validation set’ and ‘test set_A.’ This resulting dataset was then further split into ‘training with validation set (n = 253)’ and ‘test set_A (n = 52)’. In the training with validation set, the results of the minor group (i.e. CJD patients; n = 37) were replicated five times (500%) to achieve a 1:1 ratio between the CJD and non-CJD groups (n = 216). Subsequently, the training with the validation set was split into separate training and validation sets with a 9:1 ratio, respectively.

### ML and DL analysis

ML analysis was performed using Waikato Environment for Knowledge Analysis 3.8.2 (WEKA) [39]. Evaluation of the J48 decision tree (confidence factor = 0.25, minimal number of objects = 2), naïve Bayes (the number of decimal places = 2), and the random forest classifier (the number of trees = 100) were performed using a default setting. Support vector machine (SVM) with sequential minimal optimization (SMO) evaluation was performed using default settings (C value = 1) with radial basis function kernel (gamma = 10). The Keras (http://keras.io) neural network library and the TensorFlow (https://www.tensorflow.org) software library were used for deep neural network (DNN) construction. Specifically, we used a multi-layer feed-forward artificial neural network with the standard back-propagation algorithm to perform binary classification [40,41]. For each DNN in the resulting ensemble, multiple hyper-parameters were adjusted, including the number of hidden layers, number of neurons in each layer, choice of activation function, choice of optimization method, and regularization techniques.

The best DNN structure consisted of one input, five hidden, and one output layers, with 20, 40, 30, 20 and 12 hidden unit numbers of each hidden layer, respectively. Ultimately, two DNNs were used to discriminate between CJD and non-CJD patients. All of the layers were subjected to a kernel initializer with the ‘random_normal’ option for normalization of each layer. ReLu [42,43] was used as the activation function in each hidden layer, and softmax was used in the last layer. The loss function was binary cross-entrophy [44], and Nesterov Adam (NAdam) [45] with default values (learning rate = 0.002, beta_1 = 0.9, and beta_2 = 0.999) was used as the optimizer of the loss function. Dropout [46] was used to overcome overfitting with a probability of 0.1 after each layer. Neural networks were constructed using the NN-SVG tool (alexlenail.me/NN-SVG/index.html). The best DNN structure was also used for two- or three-variable combination analysis from 6 variables. Therefore, 35 combinations were tested to find the best combination set for improving the discrimination accuracy and identifying potential ‘landmark CSF markers’.

### Statistical analysis

The performance of the ML and DL was evaluated according to the quantitative parameters of the true positive (TP), true negative (TN), false positive (FP), and false negative (FN) rates, which were then used to calculate the accuracy, sensitivity, and specificity according to the following equations:

Accuracy = (TP + TN)/(TP + TN + FP + FN)

Sensitivity = TP/(TP + FN)

Specificity = TN/(TN + FP)

The area under the receiver operating characteristic curve (AUC) was calculated to determine the best model.

### 10-fold cross-validation classification performance

Cross-validation estimation was performed using the oversampled dataset which used in ML and DL analysis to determine the predictive performance of the model and for tuning hyper-parameters as described previously [47,48]. In brief, the oversampled dataset excluding test set_A (n = 52) was split into 10 subsets. A repetition consisted of 10 iterations, and one fold was used as validation data for each iteration, while the other folds were used during training performance. After the initial training period, the performance of the network was analysed based on the validation data for tuning hyper-parameters. The training process was repeated 50 times to obtain a stable result. Each iteration was performed independently, so that each iteration had no prior knowledge about the chosen learning models in the other iterations.

## Results

**Figure 1.**
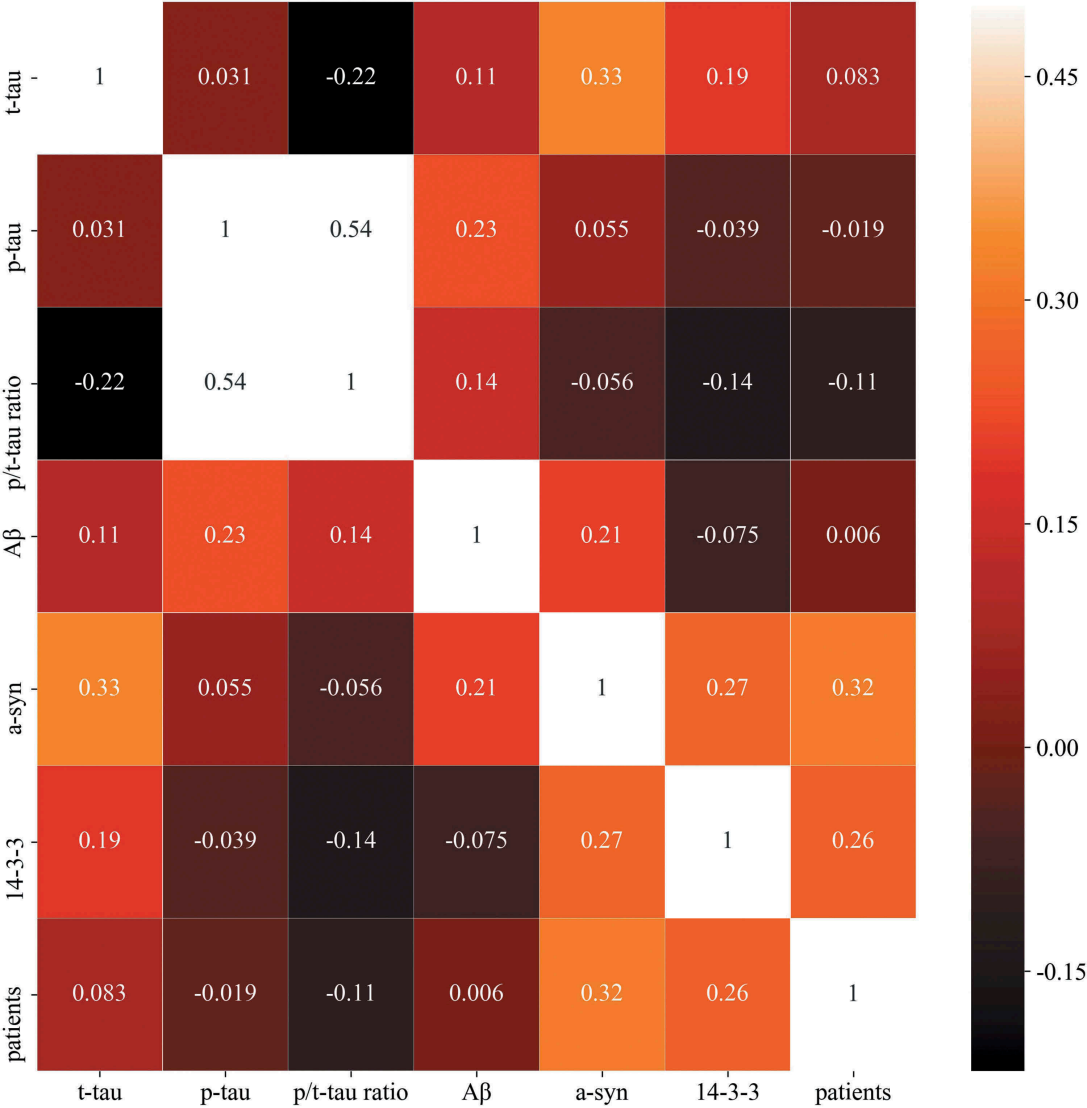
Heat map of the correlation of t-tau, p-tau, p/t-tau ratio, Aβ, a-syn and 14–3-3 levels with patients. The colour of each square depicts the correlation level, ranging from black (negative correlation) to red (intermediate correlation value) to white (positive correlation value).

**Figure 2.**
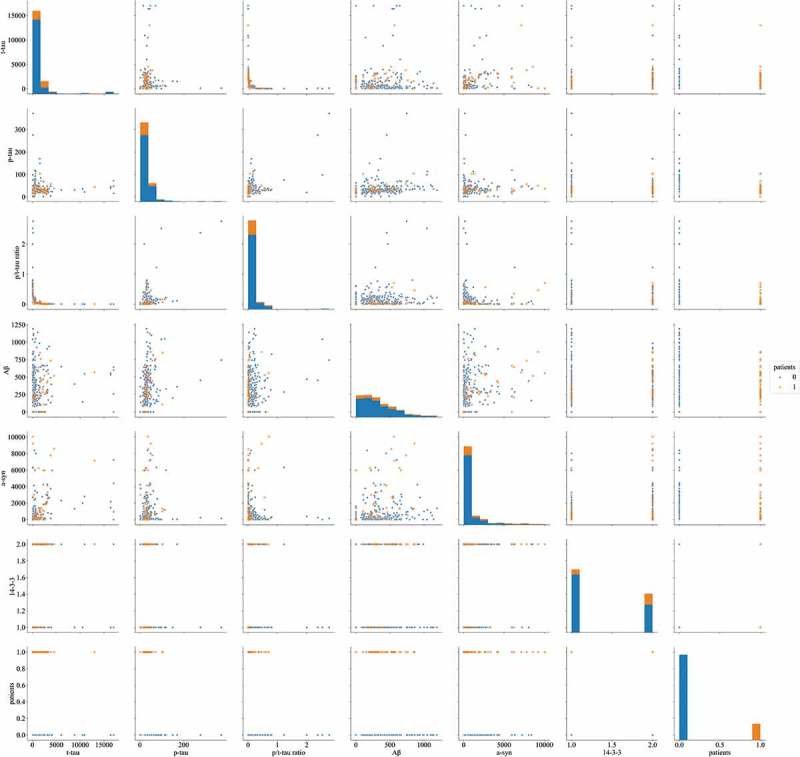
Discrimination plot for discrimination between CJD (orange) and non-CJD (blue) patients based on values of six CSF biomarkers.

The concentrations of six CSF markers (14–3-3, t-tau, p-tau, p/t-tau ratio, Aβ, and a-syn) in 5 definite, 44 probable, 11 possible CJD patients, and 256 non-CJD patients, which were determined using ELISA and western blotting, are summarized in Supplementary Table 1. A correlation heat map among the six biomarkers and sCJD patients, except for patients with possible CJD and non-CJD patients, is presented in Figure 1. None of the biomarkers showed a particularly strong correlation. Figure 2 shows the discrimination plots of the patients in the dataset according to the six biomarkers, in which each pixel represents a given CSF protein level or 14–3-3 western blot result (positive or negative): orange pixels depict CJD patients and blue pixels represent non-CJD patients. No bivariate combination was found to effectively separate the two groups. The diagnostic performances of 14–3-3, t-tau, the p/t-tau ratio, and a-syn were assessed according to the reported diagnostic criteria (Table 2). The sensitivity and specificity of the 14–3-3 protein analysis were 67.35% and 67.58%, respectively, and the age in definite and probable CJD cases with 14–3-3 positivity (mean age 69.94 ± 10.73 years) was higher than those of cases with 14–3-3 negativity (mean age 61 ± 11.6 years, supplementary Table 2).

**Table 2.** Diagnostic performance of various cerebrospinal fluid markers for Creutzfeldt-Jakob disease according to defined criteria.

		Sensitivity % (TP/all)		Specificity % (TN/all)		
Markers	Criteria	Definite and probable	Possible	Non-CJD	Neuronal diseases	References
14–3-3	14–3-3 positive^a^	67.35 (33/49)	54.54 (6/11)	67.58 (173/256)	N/A	^[52]^
t-tau	> 1,000 pg/mL	59.18 (29/49)	72.73 (8/11)	77.34 (198/256)	86.67 (13/15)	^[49]^
t-tau	> 1,300 pg/mL	53.06 (26/49)	72.73 (8/11)	80.86 (207/256)	86.67 (13/15)	^[50]^
t-tau with p/t-tau ratio	t-tau > 1,000 pg/ml with p/t tau ratio < 0.04	55.1 (27/49)	72.73 (8/11)	79.3 (203/256)	93.33 (14/15)	^[49]^
Aβ	< 445 pg/mL	69.39 (34/49)	81.82 (9/11)	28.91 (74/256)	N/A	^[51]^
a-syn	> 820 pg/mL	55.1 (27/49)	27.27 (3/11)	78.91 (202/256)	93.33 (14/15)	^[10]^
a-syn	> 680 pg/mL	55.1 (27/49)	27.27 (3/11)	74.61 (191/256)	86.67 (13/15)	^[17]^

^a^Weak positive result considered as negative N/A: not analysed

The highest diagnostic specificity in non-CJD patients was 81.6% using t-tau, and in neuronal diseases patients was 93.33% using a-syn level or t-tau combination test with p/t-tau ratio. The diagnostic sensitivity in possible CJD patients using the a-syn protein was lower than that of other proteins in these patients. The concentration of t-tau, p-tau and a-syn of the test set_B are described in Supplementary Table 1.

**Figure 3.**
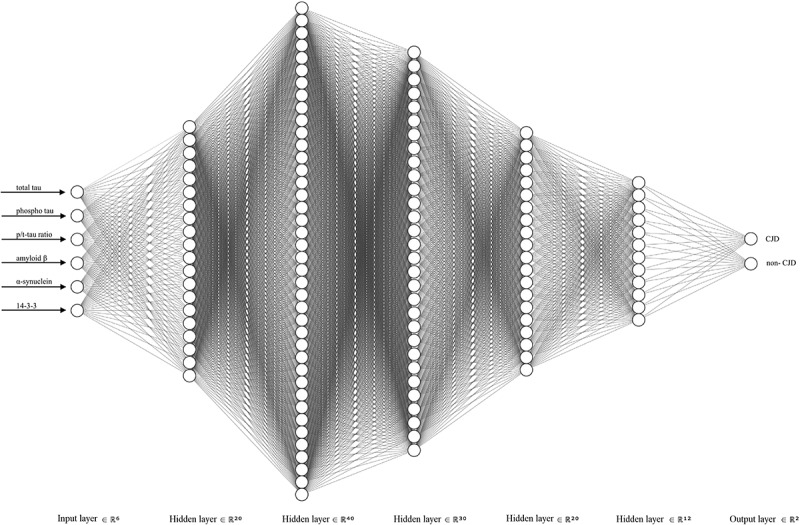
Deep neural network showing the best performance for CJD discrimination. Our network structure consisted of one input, five hidden, and one output layer. The five hidden layers consisted of 20, 40, 30, 20, and 12 hidden unit numbers of each layer, respectively. The last two output units were used to distinguish between CJD and non-CJD patients.

The performance of each classifier is described in Table 3. In the machine learning analysis using training with a validation set, the highest accuracy was observed in the analysis using J48 and the random forest classifier (78.85%). However, the machine analysis sensitivity was 41.67% or 33.33%. Although the highest specificity was observed in the analysis using the random forest classifier (92.5%), the related sensitivity was the lowest compared with analysis using the other classifiers. The best performing classifier was the DNN model, and it showed 86.54% discrimination accuracy and an AUC value of 0.90 (Figure 3).

**Table 3.** Evaluation of classifier performances (test set_A, 12 of CJD cases and 40 of non-CJD patients).

Classifier	Accuracy	Sensitivity	Specificity	AUC
SVM	76.92% (40/52)	66.67% (8/12)	80% (32/40)	0.73
Decision tree (J48)	78.85% (41/52)	41.67% (5/12)	90% (36/40)	0.8
Naïve Bayes	76.92% (40/52)	66.67% (8/12)	80% (32/40)	0.72
Random Forest	78.85% (41/52)	33.33% (4/12)	92.5% (37/40)	0.82
DNN	86.54%(45/52)	83.33%(10/12)	87.5% (35/40)	0.90

Accuracy: TP+TN/No. of cases in test set_A Sensitivity: TP/No. of CJD cases in test set_A Specificity: TN/No. of non-CJD cases in test set_A

To improve the discrimination and identify potential ‘landmark CSF markers’ for discriminating between CJD and non-CJD patients, the structure of the best-performing model described above was used for analysis with two- or three-variable combinations. As shown in Table 4, AUC is not necessarily equivalent to the accuracy measured at a threshold because an AUC score considers the sensitivity and specificity of the various threshold changes in each classification model. The best accuracy and AUC values were obtained for the three-marker combination of t-tau, p-tau, and a-syn. The scores for the test set_A were 90.38% (47/52) accuracy, 83.33% (10/12) sensitivity, and 92.5% (37/40) specificity. Using the established discrimination model, 10-fold cross-validation was performed. The validation was repeated 50 times, and the AUC and accuracy scores were calculated to obtain a stable result (Supplementary Table 3). The model was then re-evaluated using test set_B, which only consisted of CSF samples from patients with neuronal diseases, and all the samples were predicted correctly to not have CJD.

**Table 4.** Analysis of two- and three-protein combinations for discrimination between CJD and non-CJD patients. All values were calculated using the oversampled validation set and test set_A.

Combinations	Accuracy in validation set	AUC in validation set	Accuracy in test set_A	AUC in test set_A
t-tau and p-tau	70.73%	0.83	69.23%	0.71
t-tau and p/t-tau ratio	65.85%	0.77	73.08%	0.76
t-tau and Aβ	80.49%	0.91	75%	0.76
t-tau and a-syn	80.49%	0.91	82.69%	0.86
t-tau and 14–3-3	63.41%	0.80	69.23%	0.79
p-tau and p/t-tau ratio	65.85%	0.83	61.54%	0.58
p-tau and Aβ	51.22%	0.51	46.15%	0.55
p-tau and a-syn	58.54%	0.72	65.38%	0.84
p-tau and 14–3-3	65.85%	0.71	65.38%	0.60
p/t-tau ratio and Aβ	48.78%	0.46	48.08%	0.52
p/t-tau ratio and a-syn	53.66%	0.62	59.62%	0.84
p/t-tau ratio and 14–3-3	68.29%	0.77	69.23%	0.75
Aβ and a-syn	65.85%	0.76	69.23%	0.74
Aβ and 14–3-3	56.1%	0.76	38.46%	0.42
a-syn and 14–3-3	60.98%	0.69	75%	0.90
t-tau, p-tau and p/t-tau ratio	85.21%	0.85	78.85%	0.85
t-tau, p-tau and Aβ	78.05%	0.88	75%	0.74
t-tau, p-tau and a-syn	87.8%	0.95	90.38%	0.88
t-tau, p-tau and 14–3-3	78.05%	0.91	71.15%	0.71
t-tau, p/t-tau ratio and Aβ	75.61%	0.85	80.77%	0.77
t-tau, p/t-tau ratio and a-syn	75.61%	0.90	75%	0.84
t-tau, p/t-tau ratio and 14–3-3	70.73%	0.82	76.92%	0.78
t-tau, Aβ and a-syn	78.05%	0.85	86.54%	0.86
t-tau, Aβ and 14–3-3	87.8%	0.95	78.85%	0.75
t-tau, a-syn and 14–3-3	87.8%	0.98	78.85%	0.86
p-tau, p/t-tau ratio and Aβ	46.34%	0.54	51.92%	0.60
p-tau, p/t-tau ratio and a-syn	68.29%	0.72	71.15%	0.77
p-tau, p/t-tau ratio and 14–3-3	70.73%	0.84	71.15%	0.65
p-tau, Aβ and a-syn	73.17%	0.88	84.62%	0.91
p-tau, Aβ and 14–3-3	58.54%	0.75	40.38%	0.44
p-tau, a-syn and 14–3-3	65.85%	0.70	71.15%	0.74
p/t-tau ratio, Aβ and a-syn	65.85%	0.83	69.23%	0.72
p/t-tau ratio, Aβ and 14–3-3	56.1%	0.55	26.92%	0.40
p/t-tau ratio, a-syn and 14–3-3	60.98%	0.62	71.15%	0.82
Aβ, a-syn and 14–3-3	63.41%	0.76	67.31%	0.75

Accuracy: TP+TN/No. of cases in test set_A Sensitivity: TP/No. of CJD cases in test set_A Specificity: TN/No. of non-CJD cases in test set_A

## Discussion

At present, sCJD can only be diagnosed with certainty after a patient is deceased, based on histological examination of the brain tissue. Analysis of protein biomarkers related to CJD has proven to be a potentially useful alternative pre-mortem diagnosis method. The 14–3-3 protein has been reported to be a biomarker for rapid progressive neurodegenerative disorders, including CJD. In our data, analysis for CSF 14–3-3 revealed a dependency on age; the sensitivity was higher in definite and probable sCJD cases than in possible sCJD cases. However, the sensitivity and specificity of CSF 14–3-3 in sCJD cases were found to be lower than those reported previously [17,52] but were similar to those indicated in some studies in Japan [22], China [53] and USA [54,55]. The lower sensitivity and specificity in our results compared to those of other studies described above is assumed to be due to the following reasons. First, the 129 MM2 type, for which the 14–3-3 protein is reported to have relatively low sensitivity, was not excluded while calculating the sensitivity and specificity of 14–3-3 protein analysis. Second, experimental differences and/or laboratory errors could have been the contributing factors. Furthermore, 14–3-3 protein concentration could have changed in patients from the date of collection of the CSF sample to date of discrimination analysis.

The specificities of tau and a-syn analysis in neuronal disease patients, which were used as test set_B, were higher (86.67%–93.33%) than these values in the non-CJD group (74.61%–80.86%). The possibility of misdiagnosis due to similarities of symptoms could not be excluded in some cases in the non-CJD group. However, the protein concentration patterns of the non-CJD group significantly differed from those of the sCJD cases.

Here, we performed ML and DL-based analysis using combination of CSF markers as a pre-mortem diagnostic method. Our initial ML and DL model had an imbalanced learning problem owing to the lower number of CSF samples from CJD patients than that from non-CJD patients, which would result in a dominant influence of the major group on the analysis. We chose to use an oversampling method rather than an under-sampling method to resolve this imbalance and avoid losing information on the larger group (non-CJD) after drastic reduction to balance the ratio between the numbers of sCJD and non-CJD cases. In particular, an ensemble of DNNs using ELISA data of t-tau, p-tau, and a-syn outperformed (90.38% accuracy, 83.33% sensitivity and 92.5% specificity) the diagnostic performance of diagnostic performance of any single CSF marker described in Table 2 and other combination with DL analysis.

RT-QuIC analysis has been used to detect PrP^Sc^ in CSF samples directly, showing high diagnostic specificity [20,21,56]. However, the sensitivity of this method might rely on the specific PrP^Sc^ concentration in each CSF sample. Thus, cases in which the PrP^Sc^ concentration in a CSF sample is below the detection limit of the RT-QuIC assay, the DL-aided discrimination method could be an alternative pre-mortem CJD diagnosis method.

Despite the good performances of the models developed in this study, the discrimination between CJD and non-CJD patients was not completely accurate. We consider the following limitations of the proposed method that may have contributed to this result. First, noise among samples might have been derived from misdiagnosis for cases in which the symptoms of CJD are misinterpreted, since they are similar to those of other neurodegenerative disorders [57,58]. Second, additional combinations with other proteins associated with neurodegenerative disorders such as S-100 should be tested. Third, the hyper-parameters combination might not have been optimized since there is currently no established optimization method. In addition, the relatively small sample size might have limited the ability to construct a robust algorithm to effectively discriminate between CJD and non-CJD patients. Typically, deep learning analyses require an extremely large dataset; therefore, performing deep learning analysis with a relatively small dataset is a significant challenge. However, there are several methods to overcome the limitation of small sample size; we used the oversampling method [36,38] and 10-fold cross validation [47,48].

Although the DL-aided discrimination model warrants further improvement in performance and classification accuracy using stacked sample sizes via further ELISA data collection and/or extended analysis with other biomarkers for use as a pre-mortem diagnostic method, the DL-based model has several advantages for clinical application. First, it can easily handle large amounts of medical data. Second, CJD and non-CJD patients can be discriminated rapidly (within one or two days). Third, the results can be obtained consistently and reproducibly without requiring a specialist to conduct the laboratory tests. Overall, our findings could help to facilitate clinical decision-making.

## Data Availability

All data generated or analysed during this study are included in https://github.com/varamos/DNN_CJD and its supplementary files 1–10.
