# Supplementary material for: Sensitivity and specificity evaluation of multiple neurodegenerative proteins for Creutzfeldt-Jakob disease diagnosis using a deep-learning approach
Source: Prion. 2019 Jul 15;13(1):141–50. doi: 10.1080/19336896.2019.1639482 (PMC6650195; doi:10.1080/19336896.2019.1639482)
Supplement: Supplemental Material [file kprn-13-01-1639482-s001.docx]

Sensitivity and specificity evaluation of multiple neurodegenerative proteins for Creutzfeldt-Jakob disease diagnosis using a deep-learning approach

Sol Moe Lee^1,2^, Jae Wook Hyeon^1^, Soo-Jin Kim^2^, Heebal Kim^2^, Ran Noh^1^, Seonghan Kim^1^, Yeong Seon Lee^1^, Su Yeon Kim^1^

^1^Division of Bacterial Disease Research, Center for Infectious Diseases Research, Korea National Institute of Health, Centers for Disease Control and Prevention, Cheongju-si, Chungcheongbuk-do, 28159, South Korea, ^2^Department of Agricultural Biotechnology and Research Institute of Agriculture and Life Sciences, Seoul National University, Seoul, 08826, South Korea

Corresponding author: Su Yeon Kim, [tenksy@korea.kr](mailto:tenksy@korea.kr)

| **Supplementary Table 1.** Biochemical results of all CJD patients (n = 49) and non-CJD patients (n = 256). |
| --- |
| \| ID \| t-tau \| p-tau \| p/t-tau \| Aβ \| a-syn \| 14-3-3 \| patients \| set \| \| --- \| --- \| --- \| --- \| --- \| --- \| --- \| --- \| --- \| \| 1 \| 10 \| 20 \| 2 \| 469.7221 \| 381.72 \| negative \| non-CJD \| test set_A \| \| 2 \| 32 \| 2 \| 0.0625 \| 223.3551 \| 255.52 \| negative \| non-CJD \| training set \| \| 3 \| 32 \| 2 \| 0.0625 \| row \| 475.12 \| negative \| non-CJD \| training set \| \| 4 \| 32 \| 2 \| 0.0625 \| row \| 133.62 \| positive \| non-CJD \| training set \| \| 5 \| 38.8 \| 29.8988 \| 0.7706 \| 317.9804 \| 201.52 \| negative \| non-CJD \| training set \| \| 6 \| 38.8 \| 98.1158 \| 2.5288 \| 1042.391 \| row \| negative \| non-CJD \| training set \| \| 7 \| 38.8 \| 15.6 \| 0.4021 \| ` \| 79.22 \| negative \| non-CJD \| test set_A \| \| 8 \| 41 \| 15.6 \| 0.3805 \| row \| row \| positive \| probable CJD \| training set \| \| 9 \| 41 \| 33 \| 0.8049 \| 828.2049 \| 314.24 \| negative \| non-CJD \| test set_A \| \| 10 \| 42.3274 \| 29.4847 \| 0.6966 \| 220.7593 \| 36.64 \| negative \| non-CJD \| training set \| \| 11 \| 48.3772 \| 28.9521 \| 0.5985 \| 164.7193 \| row \| negative \| non-CJD \| training set \| \| 12 \| 48.3772 \| 28.8189 \| 0.5957 \| row \| row \| negative \| non-CJD \| training set \| \| 13 \| 50 \| 31.2 \| 0.624 \| row \| row \| positive \| probable CJD \| training set \| \| 14 \| 50.6169 \| 29.6375 \| 0.5855 \| 457.6647 \| 158.14 \| positive \| non-CJD \| test set_A \| \| 15 \| 52.1257 \| 36.8041 \| 0.7061 \| 558.3212 \| 10050.3 \| positive \| probable CJD \| training set \| \| 16 \| 52.9547 \| 29.8617 \| 0.5639 \| 348.0891 \| 1186.08 \| weak positive \| non-CJD \| training set \| \| 17 \| 53.1434 \| 29.8821 \| 0.5623 \| 529.8183 \| 2681.88 \| positive \| probable CJD \| test set_A \| \| 18 \| 53.7549 \| 29.0852 \| 0.5411 \| 190.1923 \| 141.72 \| positive \| non-CJD \| training set \| \| 19 \| 55.3699 \| 36.6169 \| 0.6613 \| 485.5019 \| 35.015 \| weak positive \| non-CJD \| training set \| \| 20 \| 58 \| 16 \| 0.2759 \| 82.6808 \| row \| positive \| non-CJD \| training set \| \| 21 \| 59.8029 \| 23.931 \| 0.4002 \| 498.0952 \| 185.91 \| positive \| non-CJD \| training set \| \| 22 \| 61.6043 \| 75.4903 \| 1.2254 \| 287.7676 \| 6316.5 \| positive \| non-CJD \| training set \| \| 23 \| 61.7748 \| 29.9534 \| 0.4849 \| 138.8138 \| 16.865 \| positive \| non-CJD \| test set_A \| \| 24 \| 65 \| 22 \| 0.3385 \| row \| row \| negative \| non-CJD \| training set \| \| 25 \| 67 \| 31.2 \| 0.4657 \| 333.5734 \| 225.8 \| negative \| non-CJD \| training set \| \| 26 \| 69.4248 \| 38.629 \| 0.5564 \| 445.0187 \| 405.22 \| negative \| non-CJD \| training set \| \| 27 \| 69.6389 \| 17.1497 \| 0.2463 \| 516.5101 \| 182.475 \| weak positive \| non-CJD \| test set_A \| \| 28 \| 70.323 \| 21.4489 \| 0.305 \| row \| 9.268 \| negative \| non-CJD \| training set \| \| 29 \| 72 \| 15.6 \| 0.2167 \| 349.0053 \| row \| negative \| non-CJD \| training set \| \| 30 \| 73 \| 24 \| 0.3288 \| 672.031 \| 74.94 \| negative \| non-CJD \| training set \| \| 31 \| 77.6 \| 27 \| 0.3479 \| 208.7308 \| 6224.18 \| negative \| probable CJD \| test set_A \| \| 32 \| 77.6 \| 13 \| 0.1675 \| 338.0182 \| 448.92 \| negative \| non-CJD \| training set \| \| 33 \| 77.6 \| 13 \| 0.1675 \| 92.4907 \| 87.5 \| negative \| non-CJD \| training set \| \| 34 \| 77.6 \| 13 \| 0.1675 \| 237.5946 \| 701.6 \| negative \| non-CJD \| test set_A \| \| 35 \| 82.9013 \| 19.8288 \| 0.2392 \| row \| row \| negative \| non-CJD \| training set \| \| 36 \| 83.0205 \| 21.0506 \| 0.2536 \| row \| 29.726 \| positive \| non-CJD \| training set \| \| 37 \| 84.6049 \| 27.9377 \| 0.3302 \| 510.8941 \| 548.64 \| negative \| non-CJD \| training set \| \| 38 \| 85.5259 \| 20.5859 \| 0.2407 \| 276.492 \| 2709.92 \| positive \| non-CJD \| training set \| \| 39 \| 86.002 \| 23.6398 \| 0.2749 \| 203.9796 \| 8.641 \| negative \| non-CJD \| training set \| \| 40 \| 86.1033 \| 29.7437 \| 0.3454 \| 119.2825 \| 92.16 \| positive \| non-CJD \| training set \| \| 41 \| 86.1737 \| 20.5514 \| 0.2385 \| 257.7468 \| row \| negative \| non-CJD \| training set \| \| 42 \| 86.8238 \| 29.3145 \| 0.3376 \| row \| row \| positive \| non-CJD \| training set \| \| 43 \| 87.9445 \| 20.1756 \| 0.2294 \| 271.3485 \| row \| negative \| non-CJD \| training set \| \| 44 \| 88.0826 \| 20.2661 \| 0.2301 \| 224.4314 \| row \| negative \| non-CJD \| test set_A \| \| 45 \| 89.8725 \| 23.6955 \| 0.2637 \| 164.4496 \| 18.043 \| positive \| non-CJD \| training set \| \| 46 \| 90.6884 \| 20.414 \| 0.2251 \| 90.1116 \| row \| negative \| non-CJD \| training set \| \| 47 \| 91 \| 31.2 \| 0.3429 \| 101.3362 \| 208.36 \| negative \| non-CJD \| training set \| \| 48 \| 91.0866 \| 21.1579 \| 0.2323 \| 347.8625 \| 316.02 \| weak positive \| non-CJD \| training set \| \| 49 \| 91.7047 \| 19.8067 \| 0.216 \| 94.8545 \| row \| negative \| non-CJD \| training set \| \| 50 \| 92.0711 \| 28.2883 \| 0.3072 \| 427.8686 \| 62.44 \| negative \| non-CJD \| test set_A \| \| 51 \| 93.3892 \| 20.4826 \| 0.2193 \| 341.0182 \| 37.26 \| negative \| non-CJD \| training set \| \| 52 \| 96.2777 \| 23.9549 \| 0.2488 \| 678.4017 \| 582.82 \| negative \| non-CJD \| training set \| \| 53 \| 98.3984 \| 35.101 \| 0.3567 \| 561.6501 \| 303.04 \| negative \| non-CJD \| training set \| \| 54 \| 99.8085 \| 20.3241 \| 0.2036 \| row \| row \| positive \| non-CJD \| training set \| \| 55 \| 100.1538 \| 28.6533 \| 0.2861 \| 255.9005 \| 146.26 \| negative \| non-CJD \| training set \| \| 56 \| 100.8803 \| 16.983 \| 0.1683 \| row \| 462.84 \| positive \| non-CJD \| training set \| \| 57 \| 102.0269 \| 22.0158 \| 0.2158 \| 200.0428 \| 63.238 \| negative \| non-CJD \| training set \| \| 58 \| 102.6299 \| 19.7293 \| 0.1922 \| 177.6193 \| 129.5 \| negative \| non-CJD \| training set \| \| 59 \| 104.3476 \| 40.706 \| 0.3901 \| 443.1809 \| 113.94 \| negative \| non-CJD \| training set \| \| 60 \| 104.5539 \| 29.1508 \| 0.2788 \| 635.1234 \| 536.12 \| negative \| non-CJD \| training set \| \| 61 \| 106.9061 \| 49.8212 \| 0.466 \| 861.2065 \| 9218 \| positive \| probable CJD \| test set_A \| \| 62 \| 109.8132 \| 43.6168 \| 0.3972 \| row \| 422.04 \| positive \| non-CJD \| training set \| \| 63 \| 111 \| 15 \| 0.1351 \| 280.8884 \| row \| negative \| non-CJD \| training set \| \| 64 \| 112.59 \| 21.5039 \| 0.191 \| row \| 98.46 \| negative \| non-CJD \| training set \| \| 65 \| 112.968 \| 29.3515 \| 0.2598 \| 315.6495 \| row \| negative \| non-CJD \| test set_A \| \| 66 \| 113.853 \| 22.7281 \| 0.1996 \| 457.0252 \| 147.62 \| negative \| non-CJD \| training set \| \| 67 \| 115 \| 31.2 \| 0.2713 \| 477.977 \| 448.92 \| negative \| non-CJD \| training set \| \| 68 \| 115.0198 \| 32.4141 \| 0.2818 \| 274.0938 \| 233.42 \| negative \| non-CJD \| training set \| \| 69 \| 116.3206 \| 276.2661 \| 2.375 \| 454.6128 \| 234.3 \| negative \| non-CJD \| training set \| \| 70 \| 116.917 \| 29.7188 \| 0.2542 \| 206.41 \| 353.86 \| positive \| probable CJD \| training set \| \| 71 \| 117.6117 \| 21.0399 \| 0.1789 \| 136.5797 \| 755.9 \| positive \| non-CJD \| training set \| \| 72 \| 117.9842 \| 31.7483 \| 0.2691 \| 389.3515 \| 165.3 \| positive \| non-CJD \| training set \| \| 73 \| 119 \| 24 \| 0.2017 \| 421.9191 \| 693.78 \| negative \| non-CJD \| training set \| \| 74 \| 119.2044 \| 22.0643 \| 0.1851 \| 237.6073 \| 151.16 \| weak positive \| non-CJD \| training set \| \| 75 \| 120.4752 \| 23.7947 \| 0.1975 \| 122.5407 \| 334.92 \| negative \| non-CJD \| training set \| \| 76 \| 121.3884 \| 27 \| 0.2224 \| 383.7014 \| row \| negative \| non-CJD \| test set_A \| \| 77 \| 122 \| 28 \| 0.2295 \| 300.8382 \| row \| negative \| non-CJD \| test set_A \| \| 78 \| 126.192 \| 31.938 \| 0.2531 \| 661.3776 \| 103.32 \| positive \| non-CJD \| training set \| \| 79 \| 131.7067 \| 22.4376 \| 0.1704 \| 217.3492 \| 248.08 \| negative \| non-CJD \| training set \| \| 80 \| 131.7752 \| 28.1667 \| 0.2137 \| 199.3427 \| 118.18 \| weak positive \| non-CJD \| training set \| \| 81 \| 134.6133 \| 372.0037 \| 2.7635 \| 744.8836 \| 146.16 \| negative \| non-CJD \| test set_A \| \| 82 \| 134.8385 \| 20.9694 \| 0.1555 \| 353.0709 \| 103.06 \| negative \| non-CJD \| training set \| \| 83 \| 137.6717 \| 30.1287 \| 0.2188 \| 1191.41 \| 1373.76 \| negative \| non-CJD \| training set \| \| 84 \| 139 \| 32 \| 0.2302 \| 425.9372 \| 272.38 \| negative \| non-CJD \| training set \| \| 85 \| 141.4182 \| 33.614 \| 0.2377 \| 421.4045 \| 17.011 \| negative \| non-CJD \| training set \| \| 86 \| 145.0436 \| 29.0369 \| 0.2002 \| 225.4949 \| 444.6 \| negative \| non-CJD \| training set \| \| 87 \| 146.6802 \| 32.8778 \| 0.2241 \| row \| 13.745 \| weak positive \| non-CJD \| training set \| \| 88 \| 149 \| 39 \| 0.2617 \| 238.0502 \| 614.52 \| negative \| non-CJD \| training set \| \| 89 \| 150.2342 \| 28.1667 \| 0.1875 \| 153.0133 \| row \| positive \| non-CJD \| training set \| \| 90 \| 153.7157 \| 22.8684 \| 0.1488 \| 328.7226 \| 257.22 \| weak positive \| non-CJD \| test set_A \| \| 91 \| 156 \| 17 \| 0.109 \| 570.028 \| row \| negative \| non-CJD \| training set \| \| 92 \| 157.8604 \| 33.4574 \| 0.2119 \| 736.4736 \| 641.5 \| negative \| non-CJD \| training set \| \| 93 \| 160 \| 24 \| 0.15 \| 303.9185 \| 1066.22 \| positive \| non-CJD \| training set \| \| 94 \| 160.0043 \| 23.2293 \| 0.1452 \| 292.0894 \| 182.18 \| negative \| non-CJD \| training set \| \| 95 \| 161.4993 \| 29.2258 \| 0.181 \| 240.0147 \| 7.824 \| negative \| non-CJD \| test set_A \| \| 96 \| 162.0783 \| 29.3515 \| 0.1811 \| 298.8149 \| 910.34 \| negative \| non-CJD \| training set \| \| 97 \| 162.4794 \| 24.0893 \| 0.1483 \| 491.6896 \| 502.48 \| negative \| non-CJD \| test set_A \| \| 98 \| 165 \| 24 \| 0.1455 \| 188.6209 \| 107.66 \| weak positive \| non-CJD \| training set \| \| 99 \| 169.7279 \| 46 \| 0.271 \| 350 \| 11.485 \| negative \| non-CJD \| training set \| \| 100 \| 170 \| 29 \| 0.1706 \| 313.2701 \| row \| negative \| non-CJD \| training set \| \| 101 \| 177.0043 \| 19.5644 \| 0.1105 \| 332.0869 \| 10.92 \| weak positive \| non-CJD \| training set \| \| 102 \| 179 \| 31.3147 \| 0.1749 \| 212.7297 \| 24.08 \| negative \| non-CJD \| training set \| \| 103 \| 182 \| 36 \| 0.1978 \| 836.3845 \| 1248.76 \| negative \| non-CJD \| training set \| \| 104 \| 182 \| 26 \| 0.1429 \| 151.8749 \| 137.12 \| negative \| non-CJD \| test set_A \| \| 105 \| 184 \| 28 \| 0.1522 \| row \| row \| negative \| non-CJD \| training set \| \| 106 \| 184.2341 \| 32.8204 \| 0.1781 \| 581.8687 \| 568.22 \| negative \| non-CJD \| test set_A \| \| 107 \| 195.8054 \| 29.1861 \| 0.1491 \| 202.6586 \| 13.283 \| negative \| non-CJD \| training set \| \| 108 \| 200.2337 \| 32.7321 \| 0.1635 \| 470.5847 \| 272.6 \| negative \| non-CJD \| training set \| \| 109 \| 202 \| 53 \| 0.2624 \| 1099.089 \| 841.06 \| negative \| non-CJD \| training set \| \| 110 \| 206 \| 36 \| 0.1748 \| 863.8409 \| 137.5 \| negative \| non-CJD \| training set \| \| 111 \| 208.8419 \| 49.0117 \| 0.2347 \| 551.6069 \| 368.94 \| weak positive \| non-CJD \| training set \| \| 112 \| 213 \| 49 \| 0.23 \| 760.83 \| 955.2 \| negative \| non-CJD \| training set \| \| 113 \| 215 \| 32 \| 0.1488 \| row \| row \| positive \| non-CJD \| training set \| \| 114 \| 215 \| 42.7531 \| 0.1989 \| 458.5085 \| 297.2 \| negative \| non-CJD \| training set \| \| 115 \| 218.8161 \| 32.777 \| 0.1498 \| 202.5742 \| 270.08 \| positive \| non-CJD \| training set \| \| 116 \| 222.2479 \| 24.7839 \| 0.1115 \| row \| 14.872 \| weak positive \| non-CJD \| training set \| \| 117 \| 225.7126 \| 29.3547 \| 0.1301 \| 233.12 \| 88.74 \| positive \| probable CJD \| training set \| \| 118 \| 227 \| 38 \| 0.1674 \| 218.6125 \| 137.1 \| negative \| non-CJD \| training set \| \| 119 \| 227.5832 \| 17.5131 \| 0.077 \| row \| 1613.3 \| positive \| non-CJD \| training set \| \| 120 \| 228 \| 25 \| 0.1096 \| 248.5158 \| 51.62 \| negative \| non-CJD \| training set \| \| 121 \| 228 \| 46 \| 0.2018 \| 1120.861 \| 478.38 \| negative \| non-CJD \| test set_A \| \| 122 \| 228.9467 \| 33.7323 \| 0.1473 \| 108.9799 \| 318.76 \| negative \| non-CJD \| training set \| \| 123 \| 236.1299 \| 31.7141 \| 0.1343 \| row \| 11.969 \| negative \| non-CJD \| training set \| \| 124 \| 238.0569 \| 29.3515 \| 0.1233 \| 312.4442 \| 87.5 \| negative \| non-CJD \| training set \| \| 125 \| 238.1423 \| 38.6724 \| 0.1624 \| 621.9044 \| 475.12 \| negative \| non-CJD \| training set \| \| 126 \| 239.1036 \| 22.0262 \| 0.0921 \| 185.8094 \| 7.963 \| negative \| non-CJD \| training set \| \| 127 \| 251 \| 49 \| 0.1952 \| 314.5493 \| 530.28 \| negative \| probable CJD \| training set \| \| 128 \| 254 \| 31.2 \| 0.1228 \| row \| 544.38 \| positive \| non-CJD \| training set \| \| 129 \| 254 \| 36 \| 0.1417 \| 498.6899 \| 235.04 \| negative \| non-CJD \| training set \| \| 130 \| 255 \| 37 \| 0.1451 \| 593.9685 \| 326.58 \| negative \| non-CJD \| training set \| \| 131 \| 261 \| 23 \| 0.0881 \| 126.1955 \| row \| negative \| non-CJD \| training set \| \| 132 \| 261 \| 31.2 \| 0.1195 \| 274.2706 \| 201.52 \| negative \| non-CJD \| test set_A \| \| 133 \| 263 \| 18 \| 0.0684 \| row \| row \| negative \| non-CJD \| training set \| \| 134 \| 264.5289 \| 21.8469 \| 0.0826 \| row \| 6.519 \| positive \| non-CJD \| test set_A \| \| 135 \| 272 \| 35 \| 0.1287 \| 337.9531 \| 1523.2 \| negative \| non-CJD \| training set \| \| 136 \| 275.2189 \| 29.6781 \| 0.1078 \| 106.5677 \| 99.6 \| negative \| non-CJD \| training set \| \| 137 \| 296.1102 \| 2 \| 0.0068 \| 227.3751 \| 2992.2 \| negative \| probable CJD \| training set \| \| 138 \| 297 \| 42.2057 \| 0.1421 \| 290.9078 \| 4133.64 \| positive \| non-CJD \| training set \| \| 139 \| 305.2146 \| 33.6413 \| 0.1102 \| 216.4615 \| 233.1 \| negative \| non-CJD \| test set_A \| \| 140 \| 312 \| 27 \| 0.0865 \| 530.5964 \| 158.54 \| negative \| non-CJD \| training set \| \| 141 \| 312.0472 \| 19.6453 \| 0.063 \| row \| row \| weak positive \| non-CJD \| training set \| \| 142 \| 327 \| 53 \| 0.1621 \| 416.2288 \| 109.74 \| negative \| non-CJD \| test set_A \| \| 143 \| 327.611 \| 16.6093 \| 0.0507 \| row \| row \| negative \| definite CJD \| training set \| \| 144 \| 355 \| 47 \| 0.1324 \| 360.1726 \| 362.78 \| negative \| non-CJD \| training set \| \| 145 \| 357.483 \| 32.9796 \| 0.0923 \| 474.7686 \| 469.36 \| negative \| non-CJD \| training set \| \| 146 \| 358.6393 \| 42.8869 \| 0.1196 \| 280.426 \| 1659.74 \| positive \| non-CJD \| training set \| \| 147 \| 360 \| 35 \| 0.0972 \| 164.0134 \| 8389.22 \| positive \| non-CJD \| training set \| \| 148 \| 361.3025 \| 48.7053 \| 0.1348 \| 100.4836 \| 159.686 \| positive \| non-CJD \| training set \| \| 149 \| 362.3558 \| 2 \| 0.0055 \| 488.7987 \| row \| negative \| non-CJD \| training set \| \| 150 \| 385 \| 74 \| 0.1922 \| 155.1007 \| 161.36 \| negative \| non-CJD \| training set \| \| 151 \| 386.2961 \| 62.3837 \| 0.1615 \| row \| 118.18 \| negative \| non-CJD \| training set \| \| 152 \| 393.3666 \| 31.7812 \| 0.0808 \| 661.1702 \| row \| negative \| non-CJD \| test set_A \| \| 153 \| 394.4434 \| 35.238 \| 0.0893 \| 110.2863 \| 14.184 \| negative \| non-CJD \| training set \| \| 154 \| 397.72 \| 35 \| 0.088 \| 583.5812 \| row \| negative \| non-CJD \| training set \| \| 155 \| 398 \| 32.2669 \| 0.0811 \| 109.043 \| 2390.98 \| negative \| non-CJD \| training set \| \| 156 \| 410 \| 42 \| 0.1024 \| 277.4163 \| 359.56 \| negative \| non-CJD \| test set_A \| \| 157 \| 410.3642 \| 29.4258 \| 0.0717 \| 249.379 \| 77.045 \| positive \| non-CJD \| training set \| \| 158 \| 410.5019 \| 40.0065 \| 0.0975 \| row \| 779.72 \| negative \| non-CJD \| training set \| \| 159 \| 414.5655 \| 43.8985 \| 0.1059 \| 189.8332 \| 402.62 \| negative \| non-CJD \| training set \| \| 160 \| 424.9464 \| 46.2903 \| 0.1089 \| 78.5311 \| 243.02 \| negative \| non-CJD \| training set \| \| 161 \| 427.8305 \| 25.418 \| 0.0594 \| 215.3558 \| 318.2 \| positive \| non-CJD \| training set \| \| 162 \| 431.0153 \| 24.9571 \| 0.0579 \| 638.7815 \| 36.64 \| negative \| non-CJD \| test set_A \| \| 163 \| 432 \| 37 \| 0.0856 \| 611.4956 \| 327.92 \| negative \| non-CJD \| training set \| \| 164 \| 440.8021 \| 3.9001 \| 0.0088 \| 487.7607 \| 165.3 \| negative \| non-CJD \| training set \| \| 165 \| 454 \| 62 \| 0.1366 \| 177.1551 \| 116.84 \| negative \| non-CJD \| test set_A \| \| 166 \| 459 \| 68 \| 0.1481 \| 126.0429 \| 656.24 \| negative \| non-CJD \| training set \| \| 167 \| 460 \| 17 \| 0.037 \| 121.7988 \| 166.64 \| positive \| non-CJD \| training set \| \| 168 \| 468 \| 31.2 \| 0.0667 \| 219.7772 \| 652.56 \| positive \| non-CJD \| training set \| \| 169 \| 479.9045 \| 19.1686 \| 0.0399 \| 253.0461 \| 20.202 \| positive \| non-CJD \| training set \| \| 170 \| 482.7139 \| 23.1725 \| 0.048 \| 456.7501 \| 18.301 \| positive \| non-CJD \| training set \| \| 171 \| 484.5127 \| 81.547 \| 0.1683 \| 151.0481 \| 143.879 \| positive \| non-CJD \| training set \| \| 172 \| 485.7701 \| 30.8967 \| 0.0636 \| 1001.939 \| 1008.04 \| negative \| non-CJD \| training set \| \| 173 \| 491.0766 \| 35.164 \| 0.0716 \| 114.6282 \| 10.924 \| negative \| non-CJD \| training set \| \| 174 \| 496 \| 47 \| 0.0948 \| 123.2893 \| 380.76 \| negative \| non-CJD \| training set \| \| 175 \| 496.2681 \| 23.502 \| 0.0474 \| 152.3589 \| 18.583 \| positive \| non-CJD \| training set \| \| 176 \| 500.9285 \| 30.9647 \| 0.0618 \| 386.3782 \| 95.54 \| negative \| non-CJD \| training set \| \| 177 \| 509.9002 \| 13 \| 0.0255 \| 155.3559 \| 33.74 \| positive \| non-CJD \| training set \| \| 178 \| 515.8863 \| 68.2076 \| 0.1322 \| 742.4439 \| 910.34 \| negative \| non-CJD \| training set \| \| 179 \| 528.5765 \| 119.1842 \| 0.2255 \| 330.3335 \| 5974.8 \| negative \| non-CJD \| training set \| \| 180 \| 539.2556 \| 32 \| 0.0593 \| 790.3471 \| row \| negative \| non-CJD \| training set \| \| 181 \| 544 \| 23 \| 0.0423 \| 234.8133 \| 779.72 \| negative \| non-CJD \| test set_A \| \| 182 \| 551.5899 \| 29.5049 \| 0.0535 \| 225.1666 \| 137.08 \| positive \| non-CJD \| test set_A \| \| 183 \| 552.7384 \| 51.3319 \| 0.0929 \| 329.1712 \| 1659.74 \| negative \| non-CJD \| training set \| \| 184 \| 554.7788 \| 23.9817 \| 0.0432 \| 257.6459 \| 392.14 \| weak positive \| probable CJD \| training set \| \| 185 \| 561.3282 \| 61.4546 \| 0.1095 \| 374.761 \| 3481.62 \| positive \| non-CJD \| test set_A \| \| 186 \| 561.6412 \| 15.6 \| 0.0278 \| 151.0827 \| 356.32 \| negative \| probable CJD \| training set \| \| 187 \| 566.1991 \| 57.2387 \| 0.1011 \| 443.9898 \| 87.48 \| positive \| probable CJD \| training set \| \| 188 \| 571.153 \| 34.012 \| 0.0595 \| 219.6442 \| 937.28 \| negative \| non-CJD \| training set \| \| 189 \| 592.1194 \| 25.9289 \| 0.0438 \| 183.2606 \| 354.2 \| weak positive \| probable CJD \| training set \| \| 190 \| 597 \| 38 \| 0.0637 \| 351.5646 \| 1274.4 \| negative \| non-CJD \| training set \| \| 191 \| 597 \| 79 \| 0.1323 \| 330.4144 \| 2513.38 \| positive \| non-CJD \| test set_A \| \| 192 \| 599.6083 \| 92.5393 \| 0.1543 \| 311.5648 \| 56.805 \| negative \| non-CJD \| training set \| \| 193 \| 602.5571 \| 51.3487 \| 0.0852 \| 116.9868 \| 505.64 \| weak positive \| non-CJD \| test set_A \| \| 194 \| 603.8932 \| 63.4394 \| 0.1051 \| 397.666 \| 68.4 \| negative \| non-CJD \| training set \| \| 195 \| 606 \| 102.5722 \| 0.1693 \| 106.3038 \| 1004.98 \| positive \| probable CJD \| test set_A \| \| 196 \| 632 \| 31.2 \| 0.0494 \| 560.1118 \| 791.94 \| negative \| non-CJD \| training set \| \| 197 \| 656 \| 18 \| 0.0274 \| 643.2837 \| 1341.78 \| positive \| non-CJD \| training set \| \| 198 \| 664.3672 \| 113.8421 \| 0.1714 \| 1050.22 \| 1184.22 \| negative \| non-CJD \| training set \| \| 199 \| 665.9198 \| 31.4129 \| 0.0472 \| row \| 4.199 \| weak positive \| non-CJD \| training set \| \| 200 \| 683 \| 51 \| 0.0747 \| 210.3054 \| 631.1 \| positive \| probable CJD \| training set \| \| 201 \| 690.8645 \| 75.7166 \| 0.1096 \| 173.9782 \| 721.68 \| positive \| non-CJD \| training set \| \| 202 \| 699.0611 \| 28.9534 \| 0.0414 \| 426.3225 \| 24.098 \| positive \| non-CJD \| training set \| \| 203 \| 719.1348 \| 47.8747 \| 0.0666 \| 444.5716 \| 8026.94 \| negative \| non-CJD \| training set \| \| 204 \| 739.5958 \| 27.8321 \| 0.0376 \| row \| row \| weak positive \| non-CJD \| training set \| \| 205 \| 746.242 \| 29.6178 \| 0.0397 \| 329.5774 \| 356.32 \| negative \| non-CJD \| training set \| \| 206 \| 768.0856 \| 34.1112 \| 0.0444 \| 187.5844 \| 15.331 \| negative \| non-CJD \| training set \| \| 207 \| 769.3676 \| 34.2783 \| 0.0446 \| 248.8674 \| 255.52 \| negative \| non-CJD \| training set \| \| 208 \| 790 \| 38 \| 0.0481 \| 463.1587 \| 377.8 \| negative \| non-CJD \| training set \| \| 209 \| 814.5781 \| 26.36 \| 0.0324 \| 373.7605 \| 136.275 \| weak positive \| probable CJD \| training set \| \| 210 \| 835.7137 \| 26.5896 \| 0.0318 \| row \| 22.146 \| weak positive \| non-CJD \| training set \| \| 211 \| 888 \| 25 \| 0.0282 \| 392.7114 \| 187.9 \| negative \| probable CJD \| training set \| \| 212 \| 907 \| 46 \| 0.0507 \| 925.4932 \| 567.1 \| positive \| non-CJD \| training set \| \| 213 \| 911 \| 31.2 \| 0.0342 \| 87.8799 \| 560.84 \| weak positive \| non-CJD \| training set \| \| 214 \| 938 \| 51 \| 0.0544 \| 238.1102 \| 907.2 \| negative \| probable CJD \| training set \| \| 215 \| 947.2 \| 24.5132 \| 0.0259 \| 600.9516 \| 22.972 \| positive \| non-CJD \| test set_A \| \| 216 \| 995.3048 \| 31.9993 \| 0.0322 \| 99.3519 \| 20.681 \| positive \| non-CJD \| training set \| \| 217 \| 997.483 \| 29.3145 \| 0.0294 \| 286.1538 \| 3282.28 \| negative \| non-CJD \| training set \| \| 218 \| 999.7188 \| 20.3151 \| 0.0203 \| 159.053 \| 10.31 \| positive \| non-CJD \| training set \| \| 219 \| 1000.14 \| 33.4479 \| 0.0334 \| 99.4042 \| 714.38 \| weak positive \| non-CJD \| training set \| \| 220 \| 1028 \| 31.2 \| 0.0304 \| 240.056 \| 701.6 \| negative \| non-CJD \| training set \| \| 221 \| 1061.207 \| 49.6817 \| 0.0468 \| row \| 20.749 \| weak positive \| non-CJD \| training set \| \| 222 \| 1069.859 \| 20.3771 \| 0.019 \| 128.2412 \| 10.502 \| positive \| non-CJD \| training set \| \| 223 \| 1109.799 \| 35.6245 \| 0.0321 \| 1141.863 \| 52.067 \| weak positive \| non-CJD \| training set \| \| 224 \| 1140.713 \| 30.0441 \| 0.0263 \| 584.8723 \| 25.32 \| negative \| non-CJD \| training set \| \| 225 \| 1189 \| 36.8291 \| 0.031 \| 204.584 \| 641.86 \| negative \| probable CJD \| test set_A \| \| 226 \| 1220.357 \| 20.0438 \| 0.0164 \| row \| 39.158 \| positive \| non-CJD \| training set \| \| 227 \| 1294.477 \| 32.6362 \| 0.0252 \| 583.8506 \| row \| positive \| probable CJD \| training set \| \| 228 \| 1294.477 \| 30.6105 \| 0.0236 \| row \| 1423.04 \| negative \| non-CJD \| training set \| \| 229 \| 1294.477 \| 45.7617 \| 0.0354 \| 187.4701 \| 246.28 \| positive \| non-CJD \| test set_A \| \| 230 \| 1296.1 \| 29.3515 \| 0.0226 \| 641.7154 \| 6224.18 \| positive \| probable CJD \| training set \| \| 231 \| 1316.175 \| 33.4168 \| 0.0254 \| 316.522 \| 41.417 \| negative \| non-CJD \| training set \| \| 232 \| 1318.024 \| 34.6658 \| 0.0263 \| 382.9621 \| 21.313 \| positive \| non-CJD \| training set \| \| 233 \| 1338 \| 61 \| 0.0456 \| 815.7863 \| 1407.12 \| weak positive \| non-CJD \| test set_A \| \| 234 \| 1450.125 \| 50 \| 0.0345 \| 100.5832 \| 187.24 \| negative \| non-CJD \| training set \| \| 235 \| 1476.353 \| 45.53 \| 0.0308 \| 986.7297 \| 34.822 \| positive \| non-CJD \| training set \| \| 236 \| 1508.463 \| 22.6287 \| 0.015 \| 121.2502 \| 26.426 \| positive \| non-CJD \| training set \| \| 237 \| 1516.162 \| 170 \| 0.1121 \| 360.6397 \| 48.453 \| positive \| non-CJD \| training set \| \| 238 \| 1521 \| 33 \| 0.0217 \| 280.0441 \| 1102.58 \| positive \| non-CJD \| training set \| \| 239 \| 1535.7 \| 32.1323 \| 0.0209 \| 344.565 \| 885.22 \| negative \| definite CJD \| training set \| \| 240 \| 1539.352 \| 17.8924 \| 0.0116 \| row \| 57.515 \| positive \| probable CJD \| training set \| \| 241 \| 1539.489 \| 31.469 \| 0.0204 \| 235.0985 \| 185.887 \| positive \| non-CJD \| training set \| \| 242 \| 1550.269 \| 150 \| 0.0968 \| 201.1337 \| 68.4 \| weak positive \| non-CJD \| test set_A \| \| 243 \| 1567.701 \| 34.1112 \| 0.0218 \| 359.3128 \| 39.107 \| positive \| non-CJD \| training set \| \| 244 \| 1570.255 \| 26.5593 \| 0.0169 \| 432.0695 \| 164.615 \| positive \| non-CJD \| training set \| \| 245 \| 1584.973 \| 103.4861 \| 0.0653 \| 849.6411 \| 1324.4 \| positive \| probable CJD \| test set_A \| \| 246 \| 1640.477 \| 28.62 \| 0.0174 \| 490.123 \| 43.778 \| positive \| non-CJD \| test set_A \| \| 247 \| 1660.706 \| 20.8772 \| 0.0126 \| row \| 50.08 \| positive \| non-CJD \| training set \| \| 248 \| 1671.701 \| 25.8866 \| 0.0155 \| row \| 125.626 \| positive \| probable CJD \| training set \| \| 249 \| 1700.991 \| 25.2865 \| 0.0149 \| 101.4088 \| 204.791 \| positive \| non-CJD \| training set \| \| 250 \| 1728.781 \| 31.1959 \| 0.018 \| 645.3469 \| 7.224 \| positive \| non-CJD \| training set \| \| 251 \| 1729.329 \| 25.9229 \| 0.015 \| 263.0853 \| 318.755 \| positive \| probable CJD \| training set \| \| 252 \| 1737 \| 33.6643 \| 0.0194 \| 463.2608 \| 875.5 \| positive \| definite CJD \| training set \| \| 253 \| 1739.539 \| 84.8351 \| 0.0488 \| 232.151 \| 16.928 \| weak positive \| non-CJD \| training set \| \| 254 \| 1746.492 \| 71.1382 \| 0.0407 \| 761.5113 \| 544.789 \| positive \| probable CJD \| training set \| \| 255 \| 1828 \| 39 \| 0.0213 \| row \| 1431.14 \| negative \| probable CJD \| test set_A \| \| 256 \| 1835.4 \| 30.0173 \| 0.0164 \| 449.1746 \| 1423.04 \| negative \| probable CJD \| training set \| \| 257 \| 1868.567 \| 28.1667 \| 0.0151 \| row \| 613.64 \| positive \| non-CJD \| training set \| \| 258 \| 2043.31 \| 22.9838 \| 0.0112 \| 671.4041 \| 2763.54 \| negative \| non-CJD \| training set \| \| 259 \| 2128.989 \| 24.7681 \| 0.0116 \| 329.7385 \| 216.848 \| positive \| definite CJD \| test set_A \| \| 260 \| 2130.945 \| 32.9133 \| 0.0154 \| 285.3021 \| 1148.66 \| positive \| probable CJD \| training set \| \| 261 \| 2234 \| 48 \| 0.0215 \| 471.6096 \| 654.4 \| negative \| non-CJD \| training set \| \| 262 \| 2268.9 \| 69.3879 \| 0.0306 \| 664.2059 \| 1853.62 \| positive \| non-CJD \| training set \| \| 263 \| 2268.9 \| 15.6 \| 0.0069 \| row \| 5959.84 \| positive \| probable CJD \| training set \| \| 264 \| 2269.782 \| 2 \| 0.0009 \| 364.0519 \| 4248.92 \| positive \| non-CJD \| test set_A \| \| 265 \| 2300 \| 68 \| 0.0296 \| 610.5254 \| 1839.84 \| negative \| non-CJD \| training set \| \| 266 \| 2316.4 \| 29.751 \| 0.0128 \| row \| row \| positive \| non-CJD \| training set \| \| 267 \| 2433.885 \| 20.3797 \| 0.0084 \| 106.4633 \| 1004.44 \| negative \| probable CJD \| training set \| \| 268 \| 2511.6 \| 38.5393 \| 0.0153 \| 521.7573 \| row \| positive \| probable CJD \| training set \| \| 269 \| 2528.1 \| 36.1425 \| 0.0143 \| 660.3792 \| 5959.84 \| positive \| probable CJD \| test set_A \| \| 270 \| 2571.4 \| 38.6724 \| 0.015 \| row \| 187.24 \| negative \| non-CJD \| training set \| \| 271 \| 2649.8 \| 29.3547 \| 0.0111 \| 256.8483 \| 1853.62 \| negative \| probable CJD \| training set \| \| 272 \| 2727.827 \| 17.8266 \| 0.0065 \| row \| 89.768 \| positive \| probable CJD \| test set_A \| \| 273 \| 2742.054 \| 21.7555 \| 0.0079 \| 273.1172 \| 2046.5 \| positive \| probable CJD \| training set \| \| 274 \| 2870.3 \| 28.9521 \| 0.0101 \| 107.1489 \| 2992.2 \| positive \| non-CJD \| training set \| \| 275 \| 2941.992 \| 13 \| 0.0044 \| 583.6771 \| 1966.78 \| positive \| non-CJD \| training set \| \| 276 \| 2975.096 \| 25.418 \| 0.0085 \| 298.9943 \| 1914.38 \| positive \| probable CJD \| test set_A \| \| 277 \| 3022.319 \| 28.5219 \| 0.0094 \| 290.852 \| 2161.16 \| positive \| non-CJD \| training set \| \| 278 \| 3031.552 \| 48.5377 \| 0.016 \| 665.178 \| 3047.6 \| positive \| non-CJD \| training set \| \| 279 \| 3082.1 \| 47.4607 \| 0.0154 \| 420.4454 \| 4248.92 \| positive \| probable CJD \| training set \| \| 280 \| 3100.7 \| 47.576 \| 0.0153 \| 904.5613 \| 4249.58 \| positive \| non-CJD \| training set \| \| 281 \| 3125.3 \| 32.1478 \| 0.0103 \| 444.1244 \| 4148.7 \| positive \| probable CJD \| training set \| \| 282 \| 3146.864 \| 35 \| 0.0111 \| 272.6755 \| 2931.84 \| positive \| non-CJD \| training set \| \| 283 \| 3186.593 \| 32.3787 \| 0.0102 \| 306.9933 \| 3312.3 \| positive \| probable CJD \| training set \| \| 284 \| 3199.294 \| 27.3602 \| 0.0086 \| 318.7592 \| 2542.2 \| positive \| non-CJD \| training set \| \| 285 \| 3248.049 \| 24.7309 \| 0.0076 \| 603.3679 \| 814.36 \| negative \| non-CJD \| training set \| \| 286 \| 3306.381 \| 20.3797 \| 0.0062 \| 549.0697 \| 2585.5 \| positive \| probable CJD \| training set \| \| 287 \| 3313.881 \| 19.0997 \| 0.0058 \| 170.8171 \| 2672.16 \| positive \| non-CJD \| training set \| \| 288 \| 3503.194 \| 36.787 \| 0.0105 \| 940.6527 \| 2683.86 \| weak positive \| non-CJD \| training set \| \| 289 \| 3807.767 \| 3.9001 \| 0.001 \| 136.0977 \| 3767.68 \| positive \| non-CJD \| training set \| \| 290 \| 3807.767 \| 47.7564 \| 0.0125 \| 491.0004 \| 141.72 \| positive \| non-CJD \| training set \| \| 291 \| 3807.767 \| 57.1339 \| 0.015 \| 734.8161 \| 7790.92 \| positive \| definite CJD \| test set_A \| \| 292 \| 3922.628 \| 23.7947 \| 0.0061 \| 396.8586 \| 722.94 \| weak positive \| non-CJD \| training set \| \| 293 \| 4114.171 \| 23.2293 \| 0.0056 \| 223.8361 \| 5965.66 \| positive \| non-CJD \| test set_A \| \| 294 \| 4537.8 \| 38.2345 \| 0.0084 \| 518.615 \| 8567.92 \| positive \| probable CJD \| training set \| \| 295 \| 6035.557 \| 30.8967 \| 0.0051 \| 649.0778 \| 2300.64 \| positive \| non-CJD \| training set \| \| 296 \| 8817 \| 31 \| 0.0035 \| 399.8413 \| 1306.64 \| negative \| non-CJD \| training set \| \| 297 \| 10484 \| 36 \| 0.0034 \| 143.2584 \| 2001 \| negative \| non-CJD \| training set \| \| 298 \| 10917 \| 22 \| 0.002 \| 545.4798 \| 2801.18 \| positive \| non-CJD \| training set \| \| 299 \| 13000 \| 44 \| 0.0034 \| 566.7751 \| 7137.02 \| positive \| probable CJD \| training set \| \| 300 \| 16386 \| 45 \| 0.0027 \| 535.2805 \| 1440.16 \| negative \| non-CJD \| training set \| \| 301 \| 16386 \| 39 \| 0.0024 \| 555.7485 \| 2169.04 \| negative \| non-CJD \| training set \| \| 302 \| 17000 \| 46 \| 0.0027 \| 260.4002 \| 4404.22 \| positive \| non-CJD \| training set \| \| 303 \| 17000 \| 72 \| 0.0042 \| 644.3051 \| row \| positive \| non-CJD \| training set \| \| 304 \| 17000 \| 15.6 \| 0.0009 \| row \| 937.28 \| positive \| non-CJD \| test set_A \| \| 305 \| 17000 \| 48 \| 0.0028 \| 600.3926 \| 7233.02 \| weak positive \| non-CJD \| training set \| \| ND_1 \| 107.9143 \| 23.4709 \| 0.2175 \| N/A \| 59.88 \| N/A \| ND \| test set_B \| \| ND_2 \| 601.6373 \| 88.2057 \| 0.1466 \| N/A \| 395.28 \| N/A \| ND \| test set_B \| \| ND_3 \| 294.4277 \| 48.2311 \| 0.1638 \| N/A \| 201.96 \| N/A \| ND \| test set_B \| \| ND_4 \| 41.3041 \| 0.0000 \| 0.0000 \| N/A \| 97.62 \| N/A \| ND \| test set_B \| \| ND_5 \| 285.7165 \| 41.3818 \| 0.1448 \| N/A \| 236.36 \| N/A \| ND \| test set_B \| \| ND_6 \| 216.2470 \| 37.6583 \| 0.1741 \| N/A \| 402.42 \| N/A \| ND \| test set_B \| \| ND_7 \| 181.6317 \| 35.4030 \| 0.1949 \| N/A \| 438.32 \| N/A \| ND \| test set_B \| \| ND_8 \| 162.6580 \| 27.3932 \| 0.1684 \| N/A \| 141.98 \| N/A \| ND \| test set_B \| \| ND_9 \| 287.4458 \| 52.7303 \| 0.1834 \| N/A \| 682.9 \| N/A \| ND \| test set_B \| \| ND_10 \| 758.4074 \| 38.6477 \| 0.0510 \| N/A \| 264.8 \| N/A \| ND \| test set_B \| \| ND_11 \| 243.5639 \| 40.4941 \| 0.1663 \| N/A \| 592.08 \| N/A \| ND \| test set_B \| \| ND_12 \| 1,954.3882 \| 22.2329 \| 0.0114 \| N/A \| 1668.88 \| N/A \| ND \| test set_B \| \| ND_13 \| 65.7700 \| 18.1522 \| 0.2760 \| N/A \| 245.46 \| N/A \| ND \| test set_B \| \| ND_14 \| 170.2488 \| 37.8637 \| 0.2224 \| N/A \| 568.9 \| N/A \| ND \| test set_B \| \| ND_15 \| 2,358.6000 \| 359.6874 \| 0.1525 \| N/A \| 241.04 \| N/A \| ND \| test set_B \| |
| ND = other neuronal diseases patients group N/A = not analysed  Supplementary Table 2. Age among the patients groups according to the 14-3-3 test results   \| 14-3-3 \| Age, years \| \| \| \| --- \| --- \| --- \| --- \| \| Definite and probable sCJD patients, mean (SD) \| Possible sCJD patients, mean (SD) \| Non-CJD, mean (SD) \| \| Positive \| 69.94 (10.73) \| 60.33 (9.27) \| 64.13 (15.67) \| \| Weak positive \| 66.33 (3.51) \| - \| 65.29 (13.11) \| \| Negative \| 61 (11.6) \| 65.6 (10.31) \| 62.82 (15.31) \|   SD = standard deviation  **Supplementary Table 3.** 10-fold cross-validation classification performance using the training and validation set. A learning rate of 0.002 for the Nadam optimizer was used. The epochs, dropout, and batch size were 1600, 0.1, and 300, respectively.   \| test \| set 1 \| set 2 \| set 3 \| set 4 \| set 5 \| set 6 \| set 7 \| set 8 \| set 9 \| set 10 \| overall (SD) \| \| --- \| --- \| --- \| --- \| --- \| --- \| --- \| --- \| --- \| --- \| --- \| --- \| \| 1 \| 0.92 \| 0.84 \| 0.93 \| 0.93 \| 0.97 \| 0.89 \| 0.83 \| 0.89 \| 0.85 \| 0.92 \| 0.90 (0.04) \| \| 2 \| 0.96 \| 0.89 \| 0.94 \| 0.76 \| 0.97 \| 0.91 \| 0.96 \| 0.88 \| 0.94 \| 0.84 \| 0.91 (0.06) \| \| 3 \| 0.89 \| 0.9 \| 0.92 \| 0.91 \| 0.97 \| 0.86 \| 0.89 \| 0.94 \| 0.85 \| 0.83 \| 0.90 (0.04) \| \| 4 \| 0.92 \| 0.87 \| 0.97 \| 0.9 \| 0.94 \| 0.95 \| 0.95 \| 0.88 \| 0.83 \| 0.96 \| 0.92 (0.04) \| \| 5 \| 0.92 \| 0.86 \| 0.92 \| 0.77 \| 0.97 \| 0.87 \| 0.96 \| 0.91 \| 0.92 \| 0.94 \| 0.91 (0.06) \| \| 6 \| 0.89 \| 0.93 \| 0.93 \| 0.83 \| 0.97 \| 0.95 \| 0.93 \| 0.9 \| 0.86 \| 0.88 \| 0.91 (0.04) \| \| 7 \| 0.92 \| 0.91 \| 0.92 \| 0.83 \| 0.99 \| 0.89 \| 0.91 \| 0.94 \| 0.85 \| 0.88 \| 0.90 (0.04) \| \| 8 \| 0.9 \| 0.9 \| 0.95 \| 0.9 \| 0.97 \| 0.91 \| 0.91 \| 0.95 \| 0.79 \| 0.94 \| 0.91 (0.05) \| \| 9 \| 0.9 \| 0.9 \| 0.95 \| 0.89 \| 0.94 \| 0.91 \| 0.93 \| 0.87 \| 0.9 \| 0.93 \| 0.91 (0.02) \| \| 10 \| 0.91 \| 0.89 \| 0.94 \| 0.89 \| 0.97 \| 0.91 \| 0.88 \| 0.93 \| 0.83 \| 0.96 \| 0.91 (0.04) \| \| 11 \| 0.93 \| 0.9 \| 0.92 \| 0.77 \| 0.96 \| 0.9 \| 0.91 \| 0.96 \| 0.89 \| 0.94 \| 0.91 (0.05) \| \| 12 \| 0.97 \| 0.91 \| 0.94 \| 0.88 \| 0.95 \| 0.89 \| 0.92 \| 0.92 \| 0.85 \| 0.92 \| 0.91 (0.03) \| \| 13 \| 0.87 \| 0.9 \| 0.92 \| 0.88 \| 0.98 \| 0.92 \| 0.89 \| 0.95 \| 0.9 \| 0.96 \| 0.92 (0.03) \| \| 14 \| 0.87 \| 0.85 \| 0.9 \| 0.83 \| 0.96 \| 0.94 \| 0.93 \| 0.91 \| 0.83 \| 0.95 \| 0.90 (0.05) \| \| 15 \| 0.89 \| 0.93 \| 0.89 \| 0.87 \| 0.97 \| 0.9 \| 0.94 \| 0.89 \| 0.93 \| 0.91 \| 0.91 (0.03) \| \| 16 \| 0.95 \| 0.91 \| 0.93 \| 0.87 \| 0.98 \| 0.94 \| 0.9 \| 0.92 \| 0.9 \| 0.93 \| 0.92 (0.03) \| \| 17 \| 0.94 \| 0.89 \| 0.96 \| 0.92 \| 0.97 \| 0.95 \| 0.92 \| 0.9 \| 0.82 \| 0.91 \| 0.92 (0.04) \| \| 18 \| 0.94 \| 0.89 \| 0.96 \| 0.92 \| 0.97 \| 0.95 \| 0.92 \| 0.9 \| 0.82 \| 0.91 \| 0.90 (0.04) \| \| 19 \| 0.94 \| 0.88 \| 0.91 \| 0.86 \| 0.95 \| 0.91 \| 0.86 \| 0.94 \| 0.81 \| 0.95 \| 0.90 (0.04) \| \| 20 \| 0.89 \| 0.94 \| 0.94 \| 0.86 \| 0.92 \| 0.81 \| 0.95 \| 0.93 \| 0.89 \| 0.96 \| 0.91 (0.04) \| \| 21 \| 0.9 \| 0.86 \| 0.94 \| 0.88 \| 0.97 \| 0.9 \| 0.92 \| 0.83 \| 0.85 \| 0.85 \| 0.89 (0.04) \| \| 22 \| 0.95 \| 0.9 \| 0.9 \| 0.84 \| 0.95 \| 0.94 \| 0.9 \| 0.9 \| 0.9 \| 0.94 \| 0.91 (0.03) \| \| 23 \| 0.88 \| 0.87 \| 0.9 \| 0.92 \| 0.97 \| 0.91 \| 0.91 \| 0.95 \| 0.75 \| 0.92 \| 0.90 (0.06) \| \| 24 \| 0.93 \| 0.95 \| 0.92 \| 0.88 \| 0.97 \| 0.92 \| 0.9 \| 0.91 \| 0.83 \| 0.88 \| 0.91 (0.04) \| \| 25 \| 0.93 \| 0.89 \| 0.95 \| 0.84 \| 0.97 \| 0.91 \| 0.87 \| 0.96 \| 0.89 \| 0.88 \| 0.91 (0.04) \| \| 26 \| 0.9 \| 0.9 \| 0.93 \| 0.88 \| 0.98 \| 0.97 \| 0.93 \| 0.9 \| 0.89 \| 0.89 \| 0.92 (0.03) \| \| 27 \| 0.92 \| 0.9 \| 0.95 \| 0.79 \| 0.98 \| 0.87 \| 0.91 \| 0.93 \| 0.85 \| 0.9 \| 0.90 (0.05) \| \| 28 \| 0.91 \| 0.93 \| 0.95 \| 0.91 \| 0.96 \| 0.96 \| 0.94 \| 0.9 \| 0.91 \| 0.91 \| 0.93 (0.02) \| \| 29 \| 0.93 \| 0.88 \| 0.92 \| 0.87 \| 0.97 \| 0.95 \| 0.92 \| 0.95 \| 0.82 \| 0.87 \| 0.91 (0.04) \| \| 30 \| 0.88 \| 0.9 \| 0.89 \| 0.89 \| 0.92 \| 0.89 \| 0.92 \| 0.93 \| 0.91 \| 0.88 \| 0.90 (0.02) \| \| 31 \| 0.9 \| 0.9 \| 0.93 \| 0.89 \| 0.98 \| 0.91 \| 0.87 \| 0.93 \| 0.79 \| 0.94 \| 0.90 (0.05) \| \| 32 \| 0.88 \| 0.83 \| 0.93 \| 0.92 \| 0.96 \| 0.92 \| 0.9 \| 0.94 \| 0.83 \| 0.95 \| 0.91 (0.04) \| \| 33 \| 0.92 \| 0.88 \| 0.95 \| 0.91 \| 0.95 \| 0.91 \| 0.9 \| 0.89 \| 0.92 \| 0.91 \| 0.91 (0.02) \| \| 34 \| 0.91 \| 0.82 \| 0.92 \| 0.88 \| 0.98 \| 0.89 \| 0.9 \| 0.92 \| 0.88 \| 0.94 \| 0.90 (0.04) \| \| 35 \| 0.95 \| 0.96 \| 0.97 \| 0.86 \| 0.92 \| 0.88 \| 0.93 \| 0.91 \| 0.78 \| 0.91 \| 0.91 (0.05) \| \| 36 \| 0.87 \| 0.92 \| 0.96 \| 0.88 \| 0.9 \| 0.95 \| 0.88 \| 0.97 \| 0.89 \| 0.87 \| 0.91 (0.04) \| \| 37 \| 0.92 \| 0.92 \| 0.94 \| 0.83 \| 0.96 \| 0.9 \| 0.9 \| 0.95 \| 0.92 \| 0.96 \| 0.92 (0.04) \| \| 38 \| 0.97 \| 0.86 \| 0.95 \| 0.82 \| 0.96 \| 0.83 \| 0.95 \| 0.94 \| 0.85 \| 0.91 \| 0.90 (0.06) \| \| 39 \| 0.87 \| 0.91 \| 0.86 \| 0.85 \| 0.95 \| 0.93 \| 0.96 \| 0.84 \| 0.94 \| 0.88 \| 0.90 (0.04) \| \| 40 \| 0.87 \| 0.78 \| 0.92 \| 0.87 \| 0.96 \| 0.91 \| 0.92 \| 0.91 \| 0.89 \| 0.95 \| 0.90 (0.05) \| \| 41 \| 0.89 \| 0.92 \| 0.95 \| 0.76 \| 0.95 \| 0.88 \| 0.81 \| 0.95 \| 0.86 \| 0.95 \| 0.89 (0.06) \| \| 42 \| 0.89 \| 0.87 \| 0.95 \| 0.88 \| 0.97 \| 0.91 \| 0.93 \| 0.94 \| 0.91 \| 0.94 \| 0.92 (0.03) \| \| 43 \| 0.93 \| 0.85 \| 0.9 \| 0.88 \| 0.95 \| 0.94 \| 0.92 \| 0.9 \| 0.86 \| 0.82 \| 0.89 (0.04) \| \| 44 \| 0.94 \| 0.89 \| 0.95 \| 0.83 \| 0.93 \| 0.83 \| 0.93 \| 0.96 \| 0.84 \| 0.92 \| 0.90 (0.05) \| \| 45 \| 0.9 \| 0.89 \| 0.95 \| 0.92 \| 0.96 \| 0.96 \| 0.91 \| 0.93 \| 0.83 \| 0.95 \| 0.92 (0.04) \| \| 46 \| 0.92 \| 0.93 \| 0.94 \| 0.83 \| 0.96 \| 0.85 \| 0.9 \| 0.93 \| 0.95 \| 0.88 \| 0.91 (0.04) \| \| 47 \| 0.93 \| 0.95 \| 0.94 \| 0.86 \| 0.93 \| 0.9 \| 0.93 \| 0.95 \| 0.86 \| 0.91 \| 0.92 (0.03) \| \| 48 \| 0.85 \| 0.84 \| 0.96 \| 0.87 \| 0.95 \| 0.87 \| 0.82 \| 0.93 \| 0.91 \| 0.9 \| 0.89 (0.04) \| \| 49 \| 0.89 \| 0.89 \| 0.89 \| 0.92 \| 0.96 \| 0.85 \| 0.86 \| 0.9 \| 0.79 \| 0.89 \| 0.89 (0.04) \| \| 50 \| 0.96 \| 0.91 \| 0.88 \| 0.87 \| 0.97 \| 0.94 \| 0.86 \| 0.87 \| 0.9 \| 0.89 \| 0.90 (0.04) \|   SD = standard deviation  * The files described below are included in https://github.com/varamos/DNN_CJD |

**Supplementary file 1.** The modified data of Supplementary Table S1 used for deep learning analysis

*Supplementary file 1. original_dataset.csv*

**Supplementary file 2.** Training and validation set

*Supplementary file 2. train_validation_set.csv*

**Supplementary file 3.** Test set_A

*Supplementary file 3. test_set_A.csv*

**Supplementary file 4.** Test set_B

*Supplementary file 4. test_set_B.csv*

**Supplementary file 5.** Oversampled training and validation set

*Supplementary file 5. train_validation_set_(oversampled).csv*

**Supplementary file 6.** Python code for deep neural network model using CSF biomarkers

*Supplementary file 6. DNN_model_with_CSF_markers.ipynb*

**Supplementary file 7.** Python code for 10-fold cross-validation

*Supplementary file 7. 10-Fold_cross_validation.ipynb*

**Supplementary file 8.** Python code for model estimation

*Supplementary file 8. load_model_&_analysis.ipynb*

**Supplementary file 9.** JSON format of deep neural network model

*Supplementary file 9. json_file_for_DNN_model.json*

**Supplementary file 10.** H5 file of the network weights of deep neural network

*Supplementary file 10. h5_file_for_DNN_model.h5*
